# Relationship between Deep Marginal Elevation and Periodontal Parameters: A Systematic Review

**DOI:** 10.3390/medicina59111948

**Published:** 2023-11-03

**Authors:** Mohammed Fareed Felemban, Osama Khattak, Thani Alsharari, Abdulrahman H. Alzahrani, Kiran Kumar Ganji, Azhar Iqbal

**Affiliations:** 1 Department of Maxillofacial Surgery and Diagnostic Sciences, Faculty of Dentistry, Taif University, Taif 21944, Saudi Arabia; m.felemban@tu.edu.sa; 2 Department of Restorative Dental Sciences, College of Dentistry, Jouf University, Sakaka 72388, Saudi Arabia; dr.azhar.iqbal@jodent.org; 3 Department of Restorative and Dental Science, Faculty of Dentistry, Taif University, Taif 21944, Saudi Arabia; thani.g@tu.edu.sa; 4 Department of Prosthodontics, Faculty of Dentistry, Taif University, Taif 21944, Saudi Arabia; a.alzhrani@tu.edu.sa; 5 Department of Preventive Dentistry, College of Dentistry, Jouf University, Sakaka 72388, Saudi Arabia; 6 Department of Periodontology & Oral Implantology, Sharad Pawar Dental College, Datta Meghe Institute of Higher Education & Research, Sawangi (Meghe), Wardha 442107, India

**Keywords:** crown lengthening, dental caries, deep margin elevation, periodontal ligament, periodontium, subgingival

## Abstract

*Background and Objectives*: This review focuses on reviewing studies from the literature regarding the effects of deep margin elevation on the surrounding periodontium. *Materials and Methods*: A review of the literature was carried out using the following online databases: Embase, The Cochrane Library, MEDLINE-PubMed and Google Scholar. Our search was limited to articles from 2010 to 2023. The search terms consisted of keywords and MeSH terms, which were ‘deep margin elevation’, ‘coronal margin relocation’, ‘periodontium’ and ‘periodontal tissues’. The literature was searched thoroughly by two reviewers. Initially, the titles of the articles were extracted. After removing irrelevant and duplicate articles, abstracts were assessed for relevant articles. Finally, the reviewers analyzed full-text articles. *Results:* A total of twelve articles, including one randomized clinical trial, three systematic reviews, two prospective cohort, three case series, one a clinical study, one pilot study and one a retrospective study, were selected and analyzed. *Conclusions:* The review suggests potential benefits of Deep Margin Elevation (DME) over surgical crown lengthening due to reduced invasiveness, yet conclusive effects on periodontal tissue remain unclear, warranting further studies on clinical parameters and inflammatory biomarkers.

## 1. Introduction

The advancements in restorative dentistry have led to the idea of minimally invasive dentistry. This concept follows the philosophy of biomimetics in restorative dentistry, which focuses on the goals of preserving tissue and using esthetic and functional restorative materials similar to the natural tooth [1,2,3]. Moreover, treating extensive carious lesions compromising the interproximal contact and subgingival margins is a great challenge for the dentist, as this hinders a lot of the steps of optimum restoration. Adequate caries removal, cavity preparation, impression taking, isolation via use of a rubber dam, gingival bleeding control and proper restoration placement and removal of excess cement and biologic width violation are all factors that are compromised because of deep cervical lesions [4]. The contamination with saliva, blood or gingival crevicular fluid affects the integrity of the tooth surface and restoration margin interface, thus decreasing the longevity of the restoration. The presence of subgingival margins and overhanging restoration result in periodontal problems, as there will be increased bleeding upon probing, increased probing depth, a loss of the clinical attachment level and radiographic bone loss. In short, the treatment of teeth with subgingival defects is mainly a challenge because of lack of adequate moisture and contamination control.

Biological width is the most vital factor regarding periodontal health. Usually, while restoring the deep cervical defects, biological width is violated, leading to periodontal breakdown, inflammation, gingival recession, bone level reduction and bleeding. The biological width is defined as the dimension of the soft tissue, which is attached to the portion of the tooth coronal to the crest of the alveolar bone [5]. The clinical term “biologic width” is commonly used to describe the variable dimensions of the supracrestally attached tissues in the apico-coronal direction. These tissues are composed of the junctional epithelium and supracrestal connective tissue attachment, and it is suggested that “supracrestal tissue attachment” should replace “biologic width” to improve clarity [6]. For the ease of understanding the readers, the term biological width will be used in this paper for a better understanding of the readers not aware of this change. According to the study of Gargiulo et al., the biological width is 2.04 mm, which is the sum of epithelial and connective tissue parameters [7]. A surgical approach like surgical crown lengthening is considered as a primary procedure for maintaining the biological width. More techniques include gingivectomy, which is thought to facilitate procedural steps, relocate the margin and create a favorable environment for the periodontium. Surgical crown lengthening is associated with a lot of drawbacks, like increased treatment time, overly priced, patient discomfort, compromised dental esthetics, loss of attachment and proximity to root concavities and furcation areas [8]. The procedure of deep margin elevation (DME) involves moving subgingival margins to supragingival margins using materials to enhance the marginal strength and integrity of the restorations. Clinical dentistry aims to conserve rather than be invasive, and in some instances, minimally invasive DME can be used instead of invasive procedures like crown lengthening [9]. However, recently, using one of deep margin elevation (DME) or coronal margin relocation (CMR) has been advocated as an alternative procedure to avoid the violation of biological width in cases with subgingival defects in certain clinical situations. [10]. Furthermore, for the treatment of large cavities, direct and indirect adhesive restorations are used, and these restorations require a dry working field, which can be achieved via a less invasive approach of DME. Although recommendations suggest a conservative approach, it may not be sufficient for restoring tissue around a tooth, where a change in shape is required [9]. Hence, DME can be considered in selective cases as an alternate to surgical crown lengthening.

Deep margin elevation is defined as a procedure utilizing restorative material to a newly formed cavity margin through the elevation of the deep cavity margin [11]. A deep margin elevation procedure was first proposed by Dietschi and Spreafico in 1998. Initially, it was called coronal margin relocation, but later on, Magne and Spreafico renamed it as deep margin elevation [12]. Venuti P et al. was the first study to propose a means of classification for DME procedures [13]. Another recent classification system is proposed by Ghezzi C et al. [14], which has divided the coronal margin relocation (CMR) or DME procedure into three types (Table 1). According to the classification by Venuti P et al. [13], only class 1 involves the elevation of the deep cavity margin. The rest of the three classes consist of the exposure of the cavity margin through surgical or orthodontic techniques. Surgical extrusion is considered to be a last treatment resort because of its invasive nature, the risk of periodontal ligament damage and the requirement of root canal treatment of the teeth with mature roots. Moreover, the orthodontic forced eruption preserves pulp, but it is time consuming and requires a weekly fiberotomy procedure [8].

A lot of in vitro and in vivo studies are present in the literature regarding DME. A systematic review consisting of 12 in vitro studies showed no statistically significant differences regarding the marginal quality, microleakage and fracture strength in samples with or without the DME procedure [15]. In a study by Bresser RA et al., 120 patients were evaluated for 10 years, and the authors recorded a 95.6% survival rate in teeth with DME [16]. In another cohort study, after a follow up period of 14 years, 10 teeth treated via DME restoration were considered ideal or satisfactory [17]. 

Thus, given the satisfactory success rate of DME, a thorough analysis of current research was used to evaluate the impact of DME on the surrounding periodontal tissues, as only two recent reviews are available regarding effects of DME on the periodontium, which have not included all the present studies in the literature regarding DME and the periodontium. A dentist must understand the clinical aspects of DME with respect to periodontal parameter changes. The present review aimed at reviewing studies from the literature regarding DME’s effects on the surrounding periodontium.

## 2. Materials and Methods

### 2.1. Eligibility Criteria

Inclusion Criteria: we included published journal articles and books that met the following criteria:

- Studies conducted between 2010 and 2023;
- Articles and books directly related to the research question and objectives;
- Studies indexed in Embase, The Cochrane Library, MEDLINE-PubMed and Google Scholar.

### 2.2. Exclusion Criteria

The following types of studies were excluded from our search:

- Animal and in vitro studies;
- Conference abstracts and non-peer-reviewed sources;
- Studies not relevant to the research question;
- Studies with a high risk of bias or poor methodological quality.

### 2.3. Search Strategy

Our search strategy involved the use of keywords and MeSH terms, including ‘deep margin elevation’, ‘coronal margin relocation’, ‘periodontium’ and ‘periodontal tissues.’ We used Boolean operators ‘OR’ and ‘AND’ to refine search results. Multiple terms and synonyms were employed to ensure a comprehensive search strategy, with a focus on sensitivity. The systematic review adhered to rigorous research standards, and its methodology was pre-registered with PROSPERO, an international database for systematic reviews, using the following wide reference number: CRD42023466642. This registration ensured transparency and accountability throughout the research process. Study selection was performed in two phases. Phase 1 consisted of two separate authors identifying required titles and abstracts from the literature. In phase 2, both the reviewers applied separate strategies to the complete articles. The reviewers went through the chosen studies to look for potentially significant papers. The two authors gathered necessary information from the chosen research, and they resolved any conflicts among themselves. The studies’ characteristics were recorded along with periodontal parameters measured and details of the methods and outcome assessment.

## 3. Results

**Figure 1:**
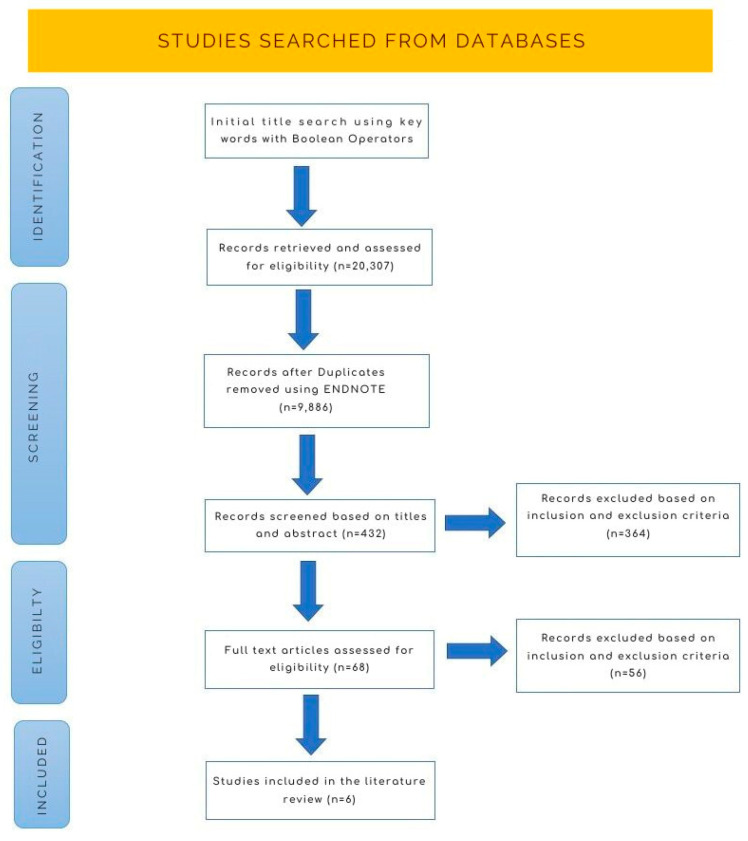
Prisma flowchart of the selection process.

The screening of the selected database yielded a total of 432 articles. Out of the total number, 364 articles were excluded after evaluation of the title and abstract. Sixty-eight articles were included for the full text assessment. The selection process resulted in a final list of six articles included in the review. Of these, three were systematic reviews, two were prospective cohorts, and one was a retrospective study (Figure 1). The characteristics and outcomes of the included studies are documented in Table 2.

## 4. Discussion

The zone of the root from the crest of the alveolar bone to the most apical part of gingival sulcus is known as biologic width, which, according to Garguilo et al., is 2.04 mm, and Vacek et al. recorded it to be 3.25 mm [7,22]. These values are not constant and may vary from case to case. For that matter, transgingival probing should be performed beforehand. When a carious lesion develops, it already violates this biologic width. As a result, a natural protective mechanism occurs in the epithelium, connective tissue and alveolar bone, in which bone resorption occurs, followed by the apical movement of the connective tissue and the epithelium, with the carious lesion lying above the bone level, thus maintaining the biological width [23]. If this biological width is violated, it leads to increased plaque accumulation and increased amounts of bacteria, which further result in gingival and periodontal inflammation. This inflammation can manifest as gingival redness, bleeding, pain, true pocket formation and clinical attachment and bone loss [5]. However, the DME restoration with smooth and highly finished surface materials might improve the environment adjacent to the bone and soft tissue, resulting in the formation of another kind of healthy biological width, with a longer junctional epithelium along the material and smaller connective tissue attachment along the remaining tooth structure below the composite [24]. Due to extensive carious lesions, the severity of the biological width violations may elicit a biological response from both hard and soft tissues [25]. The limited proximal area being infringed is more tolerable than the complete circumferential margin being infringed. A clinical trial that involved randomization showed that when subgingival proximal restorations were placed under strict plaque control, there was no significant difference in plaque index, probing depth or bleeding upon probing between the study groups after six months [26]. The results indicate that the violation of the biological width does not always equal the surgical crown lengthening. DME does not result in the re-creation of the biological width but rather a healthy variable that includes a longer junctional epithelium along with the material and a smaller connective attachment under the dentin, adjacent to the restorative material [9]. Hence, it can be concluded that the use of DME can help the clinician to maintain a healthier connective tissue in deep carious lesions. 

DME is a technique used to preserve dental structure and periodontium. Its basically a technique consisting of the application of direct resin composite upon a natural tooth structure using a pre-molded metal matrix to relocate the margin for an indirect bonded restoration. DME is an alternative to conventional surgical crown lengthening. It is indicated in the narrow and short extension of caries not more than 2 mm below the cementoenamel junction. The DME procedure is used in various deep cavity restorations, like indirect bonded restorations, indirect non-bonded restorations and direct restorations, especially those inlays and onlays that are fabricated using CAD/CAM [12]. There are many advantages of using this technique, such as their being no need for surgical procedures to relocate margins and restoration can be performed in a single session, reducing the treatment time convenient for both the patient and the clinician and, more importantly, preserving the sound tooth structure. The impression taking and luting of indirect restorations has become easier with this technique [27].

Several factors affect the success rate of the DME procedure. The clinician’s skill, adequate saliva and blood isolation, adaptation of restorative material such that to avoid secondary caries and final finishing of the restoration are some such factors [28]. After a follow-up period of 2.7 years, 70% of restorations performed with DME were given high quality ratings [29]. A knowledge, attitude and practice study regarding DME conducted in Riyadh revealed that although dentists were aware of the technique, only 30.4% have applied this in their clinical practice [30]. There is a need to emphasize this noninvasive technique from the undergraduate level. A literature review by Samartzi TK et al. constituting 44 studies, of which 24 were in vitro studies and the rest were case series and clinical studies, advocated the careful use of DME [28].

The response of gingival tissues to subgingival restorations depends on several factors, including the contour and margins of the restoration, iatrogenic issues like overhangs, marginal discrepancies and the type of restorative material. The selection of restorative material is very important in the case of DME, as a smooth, hard and clean material will result in less inflammation [31]. The connective tissue is selective in terms of the adaptation, while epithelium is not as it can easily adapt via the simple apposition of the hemidesmosomes. Therefore, material adaptation and surface texture are very important in cases of sub gingival restorations. Material chosen for DME should be of such quality that it could endure the masticatory forces and maintain marginal integrity. Many non-irritating dental materials can be used for the DME procedure. These include resin modified glass ionomers, compomers and flowable composites [32].

There are less studies in the literature discussing the effects of DME on the periodontal parameters (Table 2). Three systematic analyses were conducted on the desired impact of DME on the periodontium. The study by Mugri MH et al. assessed the prognosis of teeth restored via clinical crown lengthening and DME [18]. The systematic review included six studies, four cases if crown lengthening and two DME cases. It was concluded that DME restoration has a better survival rate compared to crown lengthening. However, the studies were highly heterogeneous, and the survival rate was not reported for the crown lengthening studies [16]. Another systematic review conducted by Chun et al. showed no changes regarding marginal bone levels, recession or clinical attachment loss. Pocket depth and bleeding upon probing increased when the distance between the restorative margin and alveolar bone was below 2 mm. Moreover, non-surgical DME procedures presented with increased pocket depth and bleeding upon, probing causing detrimental changes in the periodontium, whereas the surgical crown lengthening approach revealed some reduction in pocket depth and bleeding upon probing, making it more favorable because of fewer signs of periodontal breakdown [2]. A systematic review by Alghulikah K et al. reported that DME could be a less invasive alternative to crown lengthening and orthodontic extrusion. However, it is still not clear if DME could have a detrimental effect on the periodontium [10].

Bertoldi C et al. compared the clinical and histological responses of periodontal tissues to subgingival composite restoration and the natural root surface. The study concluded that the gingival inflammation levels at the sites where DME was performed were similar to the untreated natural root surfaces, thus making DME a favorable procedure due to optimum clinical and histological outcomes. However, they only included the sites where the distance between the cervical margin and crestal bone is 3 mm or more [19]. The study by Ferrari M et al. revealed that the DME procedure used to treat MOD cavity results in frequent bleeding upon probing with the presence of deep restoration margin [20]. Muscholl C et al. evaluated the quality of subgingival composite restoration performed via DME and the inflammatory status of the surrounding periodontal tissues. There was not any significant difference between the restored tooth and the control tooth regarding bleeding upon probing, the gingival bleeding index and the plaque control record. However, the clinical attachment level was high for the restored teeth. The quality of the restorations was regarded as good to excellent in 70% of cases. There was a positive correlation between the use of the interdental brush and the decrease in inflammation [21].

Ghezzi et al. evaluated non-surgical DME and surgical DME with the gingival and osseous approaches. A reduction in the periodontal parameters was observed after one year of the DME procedure, and no significant changes were seen in the follow-up period [14]. Furtado S et al. demonstrated no evidence of gingival inflammation or plaque accumulation at 6 months of follow-up [33]. A case series by Sarfati A et al. also reported on the favorable aspects of DME with respect to periodontal status. However, periodontal parameters were not reported [9].

DME is a technique-sensitive procedure; otherwise, it will result in restorative failure. Certain technical issues can create drawbacks in DME. These technical challenges include isolation, cementation, overhanging restoration, impression taking and clinician’s skills. Magne P et al. described eleven key elements for a successful DME procedure [12]. A matrix with a greater curve is desired; matrix stability is essential; reducing the matrix band height becomes necessary for the establishing optimum seal; to maintain proper isolation, DME can be performed before root canal treatment; an adequate matrix adaptation and seal should be ensured; the finishing of the cavity margin should be performed with sufficient water spray and fine diamond bur in order to relocate the margin; procedural steps like etching, bonding and the incremental application of material should be performed after matrix adaptation while using different composite resins; pre-heating is recommended to facilitate incremental application; to ensure the finishing of the elevated margin, the removal of any overhang and excess adhesive resin is required; to avoid any impingement on gingival tissue, performing a bitewing radiograph to rule out any overhang or slippage of resin is required; and the matrix-in-a-matrix technique is a suitable option for extremely deep and localized lesions. This technique consists of sliding a sectioned fragment of metal matrix between the margin and existing matrix [12]. Another aspect of periodontal health that has not been further explored so far is the release of resin material and other ions over time. This can happen due to erosion and degradation processes, like salivary components, chewing forces, thermal changes, chemical dietary changes and oral microorganisms [34].

## 5. Conclusions

In the light of all the studies regarding DME and periodontium in the literature, the present review concluded that some of the studies advocated the use of deep margin elevation due to its lower invasiveness compared to surgical crown lengthening. However, the effect of the DME on the periodontal tissue is still inconclusive. Further studies are needed to evaluate the periodontal clinical parameters and inflammatory biomarkers.

## Figures and Tables

**Table 1:** Classification of deep margin elevation.

Classification of DME	
Venuti P et al. [13]	Ghezzi C et al. [14]
Class 1: soft tissue retraction through rubber dam, cord and teflon	Class 1: nonsurgical CMR
Class 2: soft tissue ablation through blade, diode laser, electrosurgery and soft tissue bur	Class 2a: surgical CMR (gingival approach)
Class 3: bone and soft tissue ablation through surgical crown lengthening	Class 2b: surgical CMR (osseous approach)
Class 4: Dental tissue elevation through orthodontic extrusion, the surgical extrusion technique and the partial exodontic technique	

**Table 2:** Characteristics and recommendations of studies regarding DME.

Study	Study Design	Mean Follow Up	Material for Dme	Type of Restoration	Impact of Dme on Gingival Tissue	Recommendation
Mugri et al. [18]	Systematic review	N/a	Hybrid nanofiller composite, lithium disilicate	Indirect restoration	Better response compared to surgical crown lengthening	Inconclusive
Chun et al. [2]	Systematic review	N/a	Composites	Onlays and crowns	No change observed in clinical attachment loss, marginal bone level and recession; however, increased pocket depth and bleeding was seen upon probing	Negative
Bertoldi et al. [19]	Prospective cohort study	3 months	Composite	Crowns	Gingival inflammation levels of DME were similar to the untreated natural root surfaces	Positive
Ferrari et al. [20]	Prospective cohort study	12 months	Flow composite	Onlays	Frequent bleeding upon probing but no significant changes in plaque and gingival indices	Negative
Alghulikah et al. [10]	Systematic review	N/a			DME is well tolerated by the periodontium	Positive
Muscholl et al. [21]	Retrospective study	3 years	Composite	-	No increased periodontal or gingival inflammation was observed	Positive

## Data Availability

Data can be provided upon request to the corresponding author.
